# Ambulance Transport of the Oldest Old in Tokyo: A Population-Based Study

**DOI:** 10.2188/jea.JE20090210

**Published:** 2010-11-05

**Authors:** Yasuharu Tokuda, Toshikazu Abe, Shinichi Ishimatsu, Shigeaki Hinohara

**Affiliations:** 1 Department of Medicine, Mito Kyodo Hospital, Institute of Clinical Medicine, Graduate School of Comprehensive Human Sciences, University of Tsukuba, Ibaraki, Japan; 2 Department of Emergency and Critical Care Medicine, St. Luke’s International Hospital, Tokyo, Japan

**Keywords:** ambulance services, oldest old, symptom, admission, population-based study

## Abstract

**Background:**

Few studies have investigated ambulance utilization in people aged 85 years or older, ie, the oldest old.

**Methods:**

We conducted a 1-year population-based observational study of patients transported by ambulance to emergency departments in Tokyo, Japan, which has a population of about 12 million. Demographic data, symptoms/events associated with ambulance transport, and the proportion of hospital admissions were recorded. Transport rates by age and sex were calculated using data for the background population and ambulance transports, and the 10 most frequent symptoms/events requiring transport were compared between the oldest old and those aged 65 to 84 years.

**Results:**

Of the 642 764 patients who were transported to hospitals by ambulances, 59 570 (9%) were aged ≥85 years; 64% were women. The annual ambulance transport rate for this population was 250 per 1000/year and was significantly greater than the rate (90 per 1000/year) for those aged 65 to 84 years. The highest rate was for men aged 85 to 99 years (292 per 1000/year). Among the oldest old, the most frequent reason for ambulance transport was fall (38.5 per 1000/year), and the symptom most likely to result in hospital admission was dyspnea.

**Conclusions:**

The ambulance transport rate for the oldest old was high, particularly among men aged ≥95 years. To reduce the need for ambulance transport among the oldest old, preventive care is needed to reduce falls and acute exacerbations of cardiac and respiratory disorders.

## INTRODUCTION

Demographic forces have shaped the age composition of many industrialized countries. With an increasing proportion of older adults, Japan is a rapidly aging society.^1^ Based on life table data for 2008 from the 2010 Report of the Japanese Ministry of Health, Labour and Welfare, the average life expectancy of Japanese newborns is 86 years for females and 79 years for males; the average remaining life expectancy of Japanese at age 65 is 24 years for women and 19 years for men. The number of people aged 85 years or older (the “oldest old”) is expected to grow at a rate of over 70% between 1990 and 2020.

Because the oldest old are more likely to have multiple chronic diseases/disabilities, are at higher risk for many acute symptomatic episodes, and require greater health care resources than those aged 65 to 74 years (the young old) or 75 to 84 years (the old old), it is important to investigate the utilization of health care systems among the oldest old. However, most previous research on the utilization of health care systems among elderly adults have focused mostly on the young old or old old. The utilization patterns of the oldest old could differ from those of adults aged 65 to 84.

Among the critical components of health care systems, the ambulance service plays an important part in the continuum of health care, by providing pre-hospital care and transport in emergency situations. However, ambulance use is expensive and people who use this service have higher mortality and morbidity than walk-in patients.^2^^,^^3^ Ambulance service usage in elderly populations has not until recently been a focus of research. Several studies have examined the use of medical services by elderly patients in emergency departments,^4^^–^^8^ and others have evaluated the population-based use of emergency medical services among elderly people.^9^^–^^12^

Recent research on geriatric populations has highlighted the importance of investigating clinical and epidemiologic differences across the continuum from young old to oldest old.^13^^,^^14^ To date, there has been little analysis of the patterns of utilization of ambulance services in people aged 85 years or older, despite the rapid growth of this frail subpopulation. There could also be differences in the pattern of ambulance utilization between the oldest old, young old, and old old. The existing literature on emergency medical service use by elderly adults also does not compare the frequency of symptoms/events associated with ambulance transports among age subgroups of elderly adults. The current research aimed to address this gap by describing these patterns across a broad age range of elderly adults.

We investigated the utilization patterns of ambulance services by the oldest old in Tokyo, which has the highest number of such residents in Japan. The study was performed using a citywide pre-hospital database to evaluate population-based transport rates and to examine the most frequent symptoms/reasons for ambulance transport.

## METHODS

### Study population

The study was conducted in Tokyo, the capital of Japan, with a population of approximately 12 million in 2005, a land area of 2187 square kilometers, and a temperate climate. The Tokyo Prefectural Fire Department oversees a single-tiered system covering the entire prefecture, and basic life-support ambulances are based at 80 fire stations throughout the prefecture.^15^ Ambulance services are free of charge, which seems to encourage liberal use among Japanese, and are staffed with non-physician emergency medical technicians. Using information provided by emergency departments (EDs) in Tokyo, the ambulance staff keep a digitized record of initial medical data for all patients transported to an ED.

### Data collection

Data were reviewed for all patients transported to hospital EDs in Tokyo during the 1-year period from 1 January through 31 December 2005, using the Tokyo Fire Department’s digitized registry of data on transported patients. We included only patients who were transported to hospital EDs, including those involved in traffic accidents and fires. Multiple transports of the same patient were included.

For each patient, we collected demographic data (age and sex) and the symptom/event given as the reason for the ambulance call. Hospital admission data were collected from the ED. The confidentiality of patient data was carefully protected. No information on the address of patients was obtained, to ensure their anonymity.

We also collected data on the population of Tokyo in 2005 from official publications of the Tokyo Metropolitan Government, and population data were stratified by age and sex. Prior ethical approval of the study was obtained from the Institutional Review Board of St Luke’s International Hospital.

### Statistical analysis

The total number of ED visits requiring ambulance transport was calculated for 5-year age groups, the entire population, and each sex. Transport rates were then estimated using the population and transport numbers for each age group and sex. The ambulance transport rate among the oldest old was compared to that for residents aged 65 to 84 by estimating the exact rate ratio and its 95% confidence interval (CI).

Based on the population and transport numbers for the 10 most frequent symptoms/events, transport rates were compared between those aged 65 to 84 and those aged 85 or older. Hospital admission rates for transported patients, and frequent symptoms/events, were also compared between these populations. The chi-square test was used for comparison of binary variables. All *P* values were 2-sided and *P* < 0.05 was considered to be statistically significant. Statistical analyses were performed using SPSS 15.0J (SPSS-Japan, Tokyo, Japan).

## RESULTS

Based on the population statistics of the Tokyo Metropolitan Government, there were a total of 12 413 571 residents in Tokyo in 2005. Of these, 237 873 (2%) were aged ≥85 years and 2 055 439 (17%) were aged 65 to 84 years. Of the 642 764 patients who were transported to hospitals by ambulances in Tokyo during the 1-year study period, there were 59 570 patients aged ≥85 (9% of all patients) and 185 871 patients aged 65 to 84 (29%). The oldest patient was a man aged 99. Of those aged ≥85, 21 151 (36%) were men and 38 419 (64%) were women; of those aged 65 to 84, 95 700 (51%) were men and 90 171 (49%) were women.

Ambulance transport rates in Tokyo by age group and sex are shown in Table 1. For residents aged ≥85, the annual ambulance transport rate was 250 per 1000/year. The annual ambulance transport rate was 292 per 1000/year for men aged ≥85 and 232 per 1000/year for women aged ≥85. For residents aged 65 to 84, the annual ambulance transport rate was 90 per 1000/year. The annual ambulance transport rate was 104 per 1000/year for men aged 65 to 84 and 79 per 1000/year for women aged 65 to 84. The ambulance transport rate for the oldest old was approximately 3 times that for residents aged 65 to 84; the rate ratio was 2.78 (95% CI, 2.17–3.58). The annual ambulance transport rate for residents aged ≥85 was significantly higher than that for those aged 65 to 84 for both male and female populations. The highest rate was for men aged 85 to 99 (292 per 1000/year), and the lowest rate was for women aged 45 to 64 (31 per 1000/year); the rate for men aged 85 to 99 was more than 9 times that for women aged 45 to 64.

**Table 1.** Ambulance transport in Tokyo, by age and sex (*n* = 12 413 571)

**Overall** Age range	Transported patients /population	Transport rate (per 1000/year)	Rate ratio (95% CI)
85–99	59 570/237 873	250	reference
65–84	185 871/2 055 439	90	2.78 (2.17–3.58)
45–64	135 135/3 258 801	41	6.10 (4.37–8.70)
25–44	147 928/4 014 081	37	6.76 (4.77–9.82)
5–24	114 260/2 847 377	40	6.25 (4.46–8.96)
0–4	29 807/476 692	63	3.97 (3.00–5.32)

**Males** Age range	Transported patients /population	Transport rate (per 1000/year)	Rate ratio (95% CI)

85–99	21 151/72 432	292	reference
65–84	95 700/918 229	104	2.81 (2.24–3.55)
45–64	84 491/1 641 695	51	5.73 (4.24–7.87)
25–44	79 809/2 071 599	39	7.49 (5.35–10.75)
5–24	62 777/1 467 004	43	6.79 (4.92–9.58)
0–4	16 889/243 648	69	4.23 (3.25–5.58)

**Females** Age range	Transported patients /population	Transport rate (per 1000/year)	Rate ratio (95% CI)

85–99	38 419/165 441	232	reference
65–84	90 171/1 137 210	79	2.94 (2.27–3.84)
45–64	50 644/1 617 106	31	7.48 (5.13–11.27)
25–44	68 119/1 942 482	35	6.63 (4.63–9.75)
5–24	51 483/1 380 373	37	6.27 (4.42–9.13)
0–4	12 918/233 044	55	4.22 (3.13–5.77)

Transport rates based on the top 10 symptoms/events for the elderly population in Tokyo are shown in Table 2. Fall was the most frequent event (38.5 per 1000/year) for residents aged ≥85 and the second most frequent event (11.4 per 1000/year) for those aged 65 to 84. Pain was the second most frequent symptom (32.1 per 1000/year) for residents aged ≥85 and the most frequent event (14.6 per 1000/year) for those aged 65 to 84. Other frequent symptoms were consciousness disturbance, fever, and dyspnea. For the top 6 symptoms/events (ie, fall to weakness), transport rates for residents aged ≥85 were higher than those for residents aged 65 to 84; the rate ratios for these 6 symptoms/events were significant (*P* < 0.05).

**Table 2.** Transport rates for 10 most frequent symptoms/events among Tokyo adults aged 85 years or older

Rank	Symptom or event	Age 85 or older (*n* = 237 873)		Age 65–84 (*n* = 2 055 439)		Incidence rate ratio: a/b (95% CI)
	
		Transported patients	Transport rate per 1000/year (a)	Transported patients	Transport rate per 1000/year (b)	
1	Fall	9163	38.5	23 338	11.4	3.55 (1.89–6.63)^a^
2	Pain	7631	32.1	30 055	14.6	2.13 (1.17–3.88)^a^
3	Consciousness disturbance	7308	30.7	17 667	8.6	3.44 (1.72–6.91)^a^
4	Fever	6389	26.9	10 158	4.9	5.40 (2.31–12.6)^a^
5	Dyspnea	5866	24.7	13 491	6.6	3.57 (1.63–7.82)^a^
6	Weakness	4542	19.1	12 043	5.9	3.17 (1.33–7.55)^a^
7	Gait disturbance	1709	7.2	6024	2.9	2.33 (0.63–8.67)
8	Vomiting	1631	6.9	5275	2.6	2.33 (0.63–8.67)
9	Dizziness	1544	6.5	11 565	5.6	1.17 (0.39–3.47)
10	Nausea	1281	5.4	5502	2.7	1.67 (0.40–6.87)

^a^*P* < 0.05.

Regarding the proportions of transported patients admitted to hospital with frequent symptoms/events, those with dyspnea (87%), fever (85%), and consciousness disturbance (83%) had the highest admission rates among patients aged ≥85 (Table 3). For all 10 most frequent symptoms/events, the admission rates for residents aged ≥85 were higher than those for residents aged 65 to 84 (all *P* < 0.001).

**Table 3.** Proportions of transported patients admitted to hospital, by symptom/event

Symptom or event	Age 85 or older		Age 65–84	
Fall	4652/9163	(51%)	8051/23 338	(34%)^a^
Pain	4664/7631	(61%)	15 065/30 055	(50%)^a^
Consciousness disturbance	6079/7308	(83%)	13 142/17 667	(74%)^a^
Fever	5414/6389	(85%)	7124/10 158	(70%)^a^
Dyspnea	5076/5866	(87%)	10 146/13 491	(75%)^a^
Weakness	3627/4542	(80%)	7981/12 043	(66%)^a^
Gait disturbance	1383/1709	(81%)	4378/6024	(73%)^a^
Vomiting	1134/1631	(70%)	2915/5275	(55%)^a^
Dizziness	724/1544	(47%)	4275/11 565	(37%)^a^
Nausea	751/1281	(59%)	2310/5502	(42%)^a^

^a^*P* < 0.001 by chi-square test.

## DISCUSSION

Our study showed a considerably higher ambulance transport rate (250 per 1000/year) among the oldest old, a rate that was almost 3 times that for residents aged 65 to 84 (90 per 1000/year). These findings indicate that, among elderly adults, there is no plateau in the ambulance transport rate with respect to age. The highest transport rate was for men aged 95 to 99 (465 per 1000/year), which was almost 36 times that for girls aged 10 to 14 (13 per 1000/year). These findings, in conjunction with those of studies conducted in Yokohama City,^16^^,^^17^ suggest that ambulance services play an essential role in health care, by providing pre-hospital care to the oldest old in emergency situations. Because the population of oldest old will continue to increase rapidly in Japan and many other industrialized countries, the number of ambulance transports for the oldest old are likely to grow considerably. There will be an increase in issues related to ambulance transport of elderly patients, such as increased numbers of elderly adults involved in traffic injury and elderly adults with brain death.^18^^,^^19^

Our study is the first to focus on use of ambulance services by the oldest old; previous studies used the age category of 65 years or older to analyze utilization patterns among elderly adults.^10^^–^^12^ These studies were also conducted in communities with smaller background subpopulations of the oldest old^10^^–^^12^ and were thus unable to investigate differences in patterns of ambulance usage between the oldest old and younger elderly adults. Because Tokyo has the highest number of oldest old in the world (over 230 000), we were able to use this population composition for the purpose of our study.

Fall was identified as the most frequent reason for ambulance transport among the oldest old (38.5 per 1000/year), whereas pain was the most frequent reason for ambulance transport among those aged 65 to 84 (11.4 per 1000/year). Pain, consciousness disturbance, fever, and dyspnea were ranked second to fifth among the oldest old. Thus, since fall is the primary reason for ambulance transport among elderly adults, effective measures to prevent falls among the oldest old, including the reduction of psychotropic medication use, should be developed to reduce the number of ambulance transports.^20^^–^^22^

In our study, the highest hospital admission rates were for patients presenting with dyspnea, among those aged 85 to 99 (87%) and 65 to 84 (75%). In one study,^23^ the most frequently encountered (24%) problem among elderly adults was cardiac and respiratory disorders. Another study^12^ found that the most frequently diagnosed conditions among elderly people transported by ambulances were pulmonary and cardiac diseases. Therefore, most elderly patients presenting with dyspnea are likely to have cardiac or respiratory disorders, which are common reasons for hospital admission.^24^ Long-term care for elderly patients with cardiac or respiratory disorders is needed to prevent acute exacerbations that can frequently lead to ambulance transport and subsequent hospital admission.

Our study has several limitations. First, information on the final diagnosis was not available in our database of ambulance records. Future studies are needed to evaluate the final diagnosis in ambulance transports of the oldest old. Second, data were available for only 1 year. Third, we were unable to identify persons who used ambulance transport more than once during the study period. To measure ambulance transports, we used the number of ambulance transports to the ED as a percentage of the population by sex and age. Thus, there might have been 2 or more transports for the same person. Fourth, our study design was unable to capture condition severity among the transported patients.

Despite these limitations, our study is the first to investigate use of ambulance services and to determine the ambulance transport rate among the oldest old. Men aged 95 to 99 are at highest risk for such transports. Among the oldest old, fall was the most frequent reason for ambulance transport, and dyspnea was associated with the highest proportion of hospital admissions. Ambulance use is expensive, and people who use this service have higher mortality and morbidity than do walk-in patients. Therefore, improved care is required to prevent falls in ambulatory elderly people and to reduce acute exacerbations of cardiac and respiratory disorders. In addition, further investigations are required to evaluate the final diagnosis in ambulance transports of the oldest old.
